# Sensitivity of rapid antigen tests against SARS-CoV-2 Omicron and Delta variants

**DOI:** 10.1128/jcm.00138-23

**Published:** 2023-09-20

**Authors:** Anuradha Rao, Adrianna Westbrook, Leda Bassit, Richard Parsons, Eric Fitts, Morgan Greenleaf, Kaleb McLendon, Julie A. Sullivan, William O’Sick, Tyler Baugh, Heather B. Bowers, Filipp Frank, Ethan Wang, Mimi Le, Jennifer Frediani, Pavitra Roychoudhury, Alexander L. Greninger, Robert Jerris, Nira R. Pollock, Eric A. Ortlund, John D. Roback, Wilbur A. Lam, Anne Piantadosi

**Affiliations:** 1 The Atlanta Center for Microsystems-Engineered Point-of-Care Technologies, Atlanta, Georgia, USA; 2 Department of Pediatrics, Emory University School of Medicine, Atlanta, Georgia, USA; 3 Laboratory of Biochemical Pharmacology, Emory University, Atlanta, Georgia, USA; 4 Nell Hodgson Woodruff School of Nursing, Emory University, Atlanta, Georgia, USA; 5 Department of Pathology and Laboratory Medicine, Emory University School of Medicine, Atlanta, Georgia, USA; 6 Emory University School of Medicine, Atlanta, Georgia, USA; 7 Emory/Children’s Laboratory for Innovative Assay Development, Atlanta, Georgia, USA; 8 Department of Biochemistry, Emory University School of Medicine, Atlanta, Georgia, USA; 9 Division of Infectious Diseases, Department of Medicine, Emory University School of Medicine, Atlanta, Georgia, USA; 10 Department of Laboratory Medicine, University of Washington, Seattle, Washington, USA; 11 Children’s Healthcare of Atlanta, Atlanta, Georgia, USA; 12 Department of Laboratory Medicine, Boston Children’s Hospital and Harvard Medical School, Boston, Massachusetts, USA; 13 Aflac Cancer and Blood Disorders Center at Children’s Healthcare of Atlanta, Atlanta, Georgia, USA; 14 Wallace H. Coulter Department of Biomedical Engineering, Emory University and Georgia Institute of Technology, Atlanta, Georgia, USA; Cepheid, Shanghai, China

**Keywords:** SARS-CoV-2, rapid antigen test

## Abstract

Rapid antigen tests (RATs) have become an invaluable tool for combating the COVID-19 pandemic. However, concerns have been raised regarding the ability of existing RATs to effectively detect emerging SARS-CoV-2 variants. We compared the performance of 10 commercially available, emergency use authorized RATs against the Delta and Omicron SARS-CoV-2 variants using both individual patient and serially diluted pooled clinical samples. The RATs exhibited lower sensitivity for Omicron samples when using PCR cycle threshold (C_T_) value (a rough proxy for RNA concentration) as the comparator. Interestingly, however, they exhibited similar sensitivity for Omicron and Delta samples when using quantitative antigen concentration as the comparator. We further found that the Omicron samples had lower ratios of antigen to RNA, which offers a potential explanation for the apparent lower sensitivity of RATs for that variant when using *C*_*T*_ value as a reference. Our findings underscore the complexity in assessing RAT performance against emerging variants and highlight the need for ongoing evaluation in the face of changing population immunity and virus evolution.

## INTRODUCTION

As the SARS-CoV-2 pandemic progresses, rapid antigen tests (RATs) have become a key component of home testing, community screening, and clinical diagnostics owing to their ease of use, low cost, and speed. In the United States, there are currently 32 over-the-counter antigen tests and over 60 antigen tests that can be used at point-of-care and/or at home, available under Emergency Use Authorization (EUA), and hundreds of millions of antigen tests are used every month (1). Concurrently with these important advances in the availability, variety, and widespread use of RATs, SARS-CoV-2 continues to evolve, raising concern that new variants may harbor genetic and antigenic changes affecting test performance. The Omicron variant, which was first reported in November 2021 and quickly replaced Delta as the predominant variant in the United States, differs from Delta by seven amino acid changes and a 2-amino acid deletion in the nucleocapsid (N) protein, the target of most RATs. Prior studies have demonstrated conflicting results, ranging from decreased sensitivity of RATs for Omicron (2, 3), to comparable performance (2, 4), to higher sensitivity (5) . To better understand how RATs perform against Delta and Omicron variants of SARS-CoV-2 and how this performance is related to antigen and RNA concentration, we compared the performance characteristics of ten RATs in detecting Delta and Omicron variants. Here, we used individual clinical samples as well as standardized pools of clinical samples as test substrates. Orthogonal protein detection, RNA detection, and infectivity measurements were conducted to understand variant-specific differences in RAT results.

## MATERIALS AND METHODS

All methods are described in detail in Supplementary Materials, below are brief descriptions.

### Study design

We used sequence confirmed Delta and Omicron BA.1 individual and pooled remnant clinical samples (RCSs) to compare the performance of EUA RATs in detecting these two variants. The N protein content, PCR *C*_*T*_ values (as a rough proxy for RNA concentrations) (6, 7) and ability of individual samples to infect cells in *in vitro* infectivity assays were also measured to comprehensively evaluate differences between Delta and BA.1 variants.

### Preparation of Delta and Omicron RCS pools

As part of the NIH Variant Task Force, in collaboration with participating labs, we obtained low *C*_*T*_, sequence-verified Delta and Omicron remnant clinical samples that remained after diagnostic testing. The N2 *C*_*T*_ and N protein concentrations in these RCSs were determined at ACME POCT (The Atlanta Center for Microsystems-Engineered Point-of-Care Technologies) as part of our internal quality control (QC). The CDC N2 PCR assay *C*_*T*_ value was used as a rough proxy for RNA concentration (details in Supplementary Methods); N protein concentrations were measured by Simoa (details in Supplementary Methods). Between 4 and 21 low N2 *C*_*T*_ RCS with N protein >4,000 pg/mL were pooled to generate each Delta and Omicron pool. These pools were serially diluted, N2 *C*_*T*_ and N protein quantified, and used to compare ten EUA RATs. Each RCS pool was tested in five replicates for each RAT, except ACON and iHealth, which were tested in two replicates each due to logistical constraints. We defined the Minimum Protein Detected threshold as the lowest concentration (pg/mL) at which 100% of replicates were positive, and the Maximum N2 *C*_*T*_ Detected threshold as the highest *C*_*T*_ at which 100% of replicates were positive. In the early phase of this study, we found that testing 5 replicates yielded the same result as testing 20 replicates (Fig. S1), so we used five replicates to conserve samples and reagents. Depending on sample and reagent availability, we tested some RATs in multiple independent experiments (of two to five replicates), using a separate RCS pool or separate test lot, and calculated the Minimum Protein Detected threshold and the Maximum N2 *C*_*T*_ Detected threshold for each experiment.

### Collection and storage of individual RCS

We utilized a hospital and community-based approach for enrolling eligible COVID-19 symptomatic patients. For samples collected from July to November 2021 (Delta predominant), mid-turbinate (MT) swabs were collected in 1 mL saline and frozen at −80°C. For use in the current study, samples were thawed, 2 mL sterile saline added, frozen, re-thawed, and then analyzed by Cepheid and Quanterix assays. Subsequently, these samples were thawed and utilized for Binax and QuickVue testing and *in vitro* infectivity assays. MT swab samples collected after 7 January 2022 (Omicron predominant) were collected in 3 mL saline, analyzed fresh by Cepheid and Quanterix assays, and frozen at −80°C. After one freeze-thaw, they were used for Binax testing, QuickVue testing, and infectivity assays.

### Antigen testing using Quanterix Simoa assay

Each pool dilution and every clinical sample used in the current study was analyzed for N protein concentration using the Quanterix HD-X Simoa SARS-CoV-2 N Protein Antigen (RUO) assay (Catalog #103806), according to the manufacturer’s instructions.

### PCR testing of remnant clinical samples using Cepheid

All individual remnant clinical samples used for this study underwent PCR testing using the Cepheid GeneXpert Dx Instrument system with either Xpert Xpress CoV-2/Flu/RSV *plus* cartridges (EUA 302-6991, Rev. B., October 2021) or Xpert Xpress SARS-CoV-2 cartridges (EUA 302-3562, Rev. F January 2021) according to the manufacturer’s instructions. For the CoV-2/Flu/RSV plus assay, the resulted SARS-CoV-2 *C*_*T*_ value reflects the first of three gene targets (E, N2, or RDRP) to amplify; for the SARS-CoV-2 assay, both E and N2 *C*_*T*_ values are resulted. To determine whether the *C*_*T*_ values from the two Xpert assays could be combined for analysis, the laboratory performed a bridging study to confirm that the SARS-CoV-2 assay E target *C*_*T*_ value correlated tightly with the CoV-2 *C*_*T*_ value from the CoV-2/Flu/RSV plus assay. The samples were thawed, split, and run on both assays in parallel according to the manufacturer’s instructions (Table S4).

### SARS-CoV-2 genome sequencing

All RCS pools and individual RCSs underwent sequencing at ACME POCT, where libraries were generated using SuperScript First Strand Synthesis kit (Thermo Fisher), followed by Swift Amplicon SARS-CoV-2 Research Panel (Swift Biosciences). Illumina MiSeq was used for sequencing, and viralrecon was used for genome assembly.

### Rapid antigen test testing using pools and individual clinical samples

All rapid antigen testing (pool and individual samples) was performed blinded using the direct swab method, where the sample was spiked onto the swab and the manufacturer’s instructions were followed for testing. About 20 µL sample (as described in the IFU for BinaxNOW TM COVID-19 Ag CARD) was used for BinaxNOW, while 50 µL was used for all other RATs tested. After completion, the results were unblinded.

### Evaluation of infectious SARS-CoV-2 in individual RCS using Calu-3, Vero-TMPRSS-2, and Vero cells

For *in vitro* infectivity studies, 50 µL of each individual RCS was used to inoculate (by spinoculation) cells that were 80–90% confluent growing on a 96-well plate. After 2 h, the sample was removed, and 50 µL Opti-MEM and 150 µL methylcellulose overlay media were added. This portion of the assay was conducted in the BSL3 facility as live lab-propagated SARS-CoV-2 (Delta and BA.1) of known TCID_50/mL_ were used as positive controls. After 3–6 days of incubation (depending on the cell line used), the cells were washed with 1× PBS, fixed with chilled 1:1 methanol acetone, permeabilized with 0.2% TritonX, blocked with 1% milk, and then assayed for focus forming units (FFUs) by staining with anti-nucleocapsid antibody. Stained foci were read using an ELISpot CTL reader.

### Statistics

Some *C*_*T*_ values were above the limit of detection and were therefore set to 50, above the highest recorded *C*_*T*_ value. Likewise, some antigen concentrations were below the limit of detection by Simoa and were therefore set equal to zero (0), below the lowest detected antigen concentration. For all analyses, any observations that required imputation were removed in subsequent sensitivity analyses. To meet normality and homoskedasticity assumptions for the linear regression analysis and because there were some values set equal to 0, we used a log(*n* + 1) transformation on antigen concentrations.

We calculated the clinical sensitivity of BinaxNow and QuickVue as well as their corresponding 95% confidence intervals for Delta and Omicron samples overall and by *C*_*T*_ or antigen concentration thresholds. Clinical sensitivity was calculated by dividing the number of positive tests by the number of positive participants (samples). The sensitivity of Delta and Omicron samples on the same platform were statistically compared through chi-square or Fisher’s exact tests.

Additionally, we examined and quantified the relationship between *C*_*T*_ values and antigen concentration in COVID-19-positive samples from both the Delta and Omicron dominant eras. We calculated Pearson’s correlation coefficient between *C*_*T*_ value and antigen concentration, and performed a linear regression analysis, predicting *C*_*T*_ value from antigen concentration. In the base model, we controlled for variant status. In the full model, we additionally adjusted for vaccine status and symptom duration. Any individual who was unsure of their vaccine status in any capacity was removed from the appropriate regression analyses.

We also evaluated the association between having a positive result for Calu-3 or Vero-TMPRSS-2 culture and *C*_*T*_ value, antigen concentration (pg/mL), symptom duration, vaccine status, and age (years) through unadjusted and adjusted logistic regression analyses. There were no asymptomatic individuals included in this analysis, so symptom duration was treated as a continuous variable to better understand the relationship between days of symptoms and infectivity.

All hypotheses’ tests were two-sided and a *P* value below 0.05 was considered significant. Graphs, correlation calculations, and regression modeling were conducted in R v(4.2.0). The sensitivity of RATs and comparisons between sensitivities were calculated and conducted in R v(4.1.3). Tables were created using the gt and gtsummary package and plots were created with the ggpubr, stringr, and ggplot2 package in R (8 – 12).

## RESULTS

### Rapid antigen test sensitivities for Delta and Omicron using serially diluted, pooled clinical samples are similar when using antigen concentration as the comparator, but not when using RNA measured by cycle threshold (*C*_*T*_) value as the comparator

**Fig 1:**
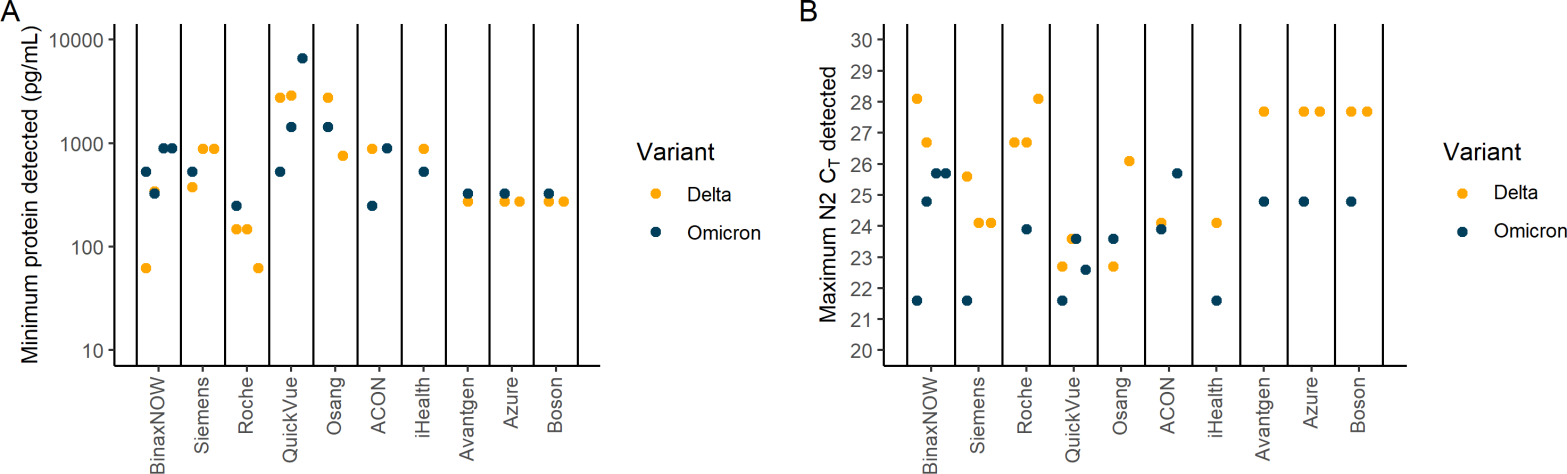
Results of testing 10 commercially available rapid antigen tests (RATs) against remnant clinical sample (RCSs) pools. Each of 10 commercially available RATs was tested using sequence-confirmed, serially diluted, and quantified (N2 C_T_ CDC Assay and Quanterix Simoa SARS-CoV-2 N Protein Antigen Test) pools generated from RCSs of the same variant. RCS pool dilutions were tested in replicate, and the Minimum Protein Detected (**A**) and Maximum N2 *C*_*T*_ Detected (**B**) were defined as the lowest and highest values at which 100% of the replicates were positive, respectively. Panels (A and B) show each RAT’s threshold, represented by an orange (Delta) or black (Omicron) filled circle. Each dot reflects one set of replicates, and when testing was repeated with an independent RCS pool, or an independent lot of tests, results are presented as separate dots. Full results from each experiment are shown in Fig. S1.

We evaluated the sensitivity of 10 commercially available RATs for Omicron and Delta using a standardized set of pooled RCSs pools that were serially diluted and quantified for SARS-CoV-2 RNA (measured by *C*_*T*_ value from CDC N2 PCR assay; described in supplementary methods) and nucleocapsid antigen concentration [measured by Quanterix Simoa Assay (13); described in supplementary methods]. Each RCS pool dilution was tested in five replicates (except ACON and iHealth which were tested in two replicates each), after confirming that this approach led to the same results as using 20 replicates (Fig. S1). For each combination of RCS pool and RAT, we defined the Minimum Protein Detected threshold as the lowest concentration (pg/mL) at which 100% of replicates were positive, and the Maximum N2 *C*_*T*_ Detected threshold as the highest *C*_*T*_ at which 100% of replicates were positive. The Minimum Protein Detected and Maximum N2 *C*_*T*_ Detected threshold for each RAT is shown in Fig. 1, and the full experimental results are shown in Fig. S2. Using the Minimum Protein Detected threshold, only the BinaxNow appeared potentially less sensitive in detecting Omicron than Delta (Fig. 1A; Fig. S2 and S3). The other tests performed similarly against Delta and Omicron pools, with a twofold or less difference in concentration (Fig. 1A; Fig. S2 and S3). However, using the Maximum N2 *C*_*T*_ Detected threshold, 7 of the 10 RATs were less sensitive in detecting Omicron than Delta (*C*_*T*_ difference ranging from 2.5 to 3.2 lower for Omicron, corresponding to a nearly 10-fold higher RNA concentration) (Fig. 1B; Fig. S2 and S3).

Thus, using RCS pools, we observed concordant sensitivity for Omicron and Delta across most commercially available RATs when measured against antigen concentration, but lower sensitivity for Omicron than Delta for over half of the RATs when measured against *C*_*T*_ value, suggesting different relationships between antigen concentration and RNA concentration for Delta versus Omicron.

### Rapid antigen test sensitivity for Delta and Omicron using individual clinical samples varies between assays and depends on choice of comparator

We next evaluated the sensitivity of two common RATs using a subset of anterior nares specimens from a large study in which 171 fresh RCSs were collected from individuals infected with Omicron and 163 banked RCS had been collected from individuals infected with Delta (Table 1; Supplementary Data File). Many of the participants were unvaccinated (40.9% and 48.5% of those infected with Delta and Omicron, respectively), and individuals infected with Omicron had shorter times since the last vaccine dose (Table 1). Per study design, most participants were symptomatic (Table 1). However, individuals infected with Omicron had shorter durations of symptoms prior to testing: nearly 80% were tested within 3 days of symptom onset and the remaining 20% within 7 days. By contrast, only about a quarter of patients with Delta were tested within 3 days of symptom onset, about half between 3 and 7 days, and about a quarter after 7 days.

**TABLE 1:** Demographics of patients with Delta (*N* = 163) and Omicron (*N* = 171)

	Delta, *n* (%)	Omicron, *n* (%)
Sex		
Female	98 (60.9%)	91 (53.2%)
Male	63 (39.1%)	80 (46.8%)
Race		
White	79 (47.9%)	80 (44.9%)
Black/African American	73 (44.2%)	75 (42.1%)
Asian	3 (1.8%)	8 (4.5%)
Other	10 (6.1%)	11 (6.2%)
Refuse to Answer	0 (0%)	4 (2.3%)
Ethnicity		
Hispanic	12 (7.4%)	26 (15.2%)
Non-Hispanic	149 (92.6%)	144 (84.2%)
Refuse to answer	0 (0%)	1 (0.6%)
Vaccine status		
Unvaccinated/not fully vaccinated	65 (40.9%)	83 (48.5%)
Fully vaccinated/boosted	94 (59.1%)	88 (51.5%)
Days since last vaccine		
Within the last 90 days	17 (17.7%)	41 (43.1%)
Between 91 and 180 days	22 (22.9%)	16 (16.8%)
Between 181 and 270 days	52 (54.2%)	28 (29.5%)
More than 270 days	5 (5.2%)	10 (10.5%)
Symptom status		
Asymptomatic	5 (3.1%)	1 (0.6%)
Symptomatic	156 (96.9%)	170 (99.4%)
Symptom duration		
Symptoms for at most 3 days	36 (23.1%)	132 (77.7%)
Symptoms for between 4 and 7 days	80 (51.3%)	38 (22.3%)
Symptoms for more than 7 days	40 (25.6%)	0 (0%)

**Fig 2:**
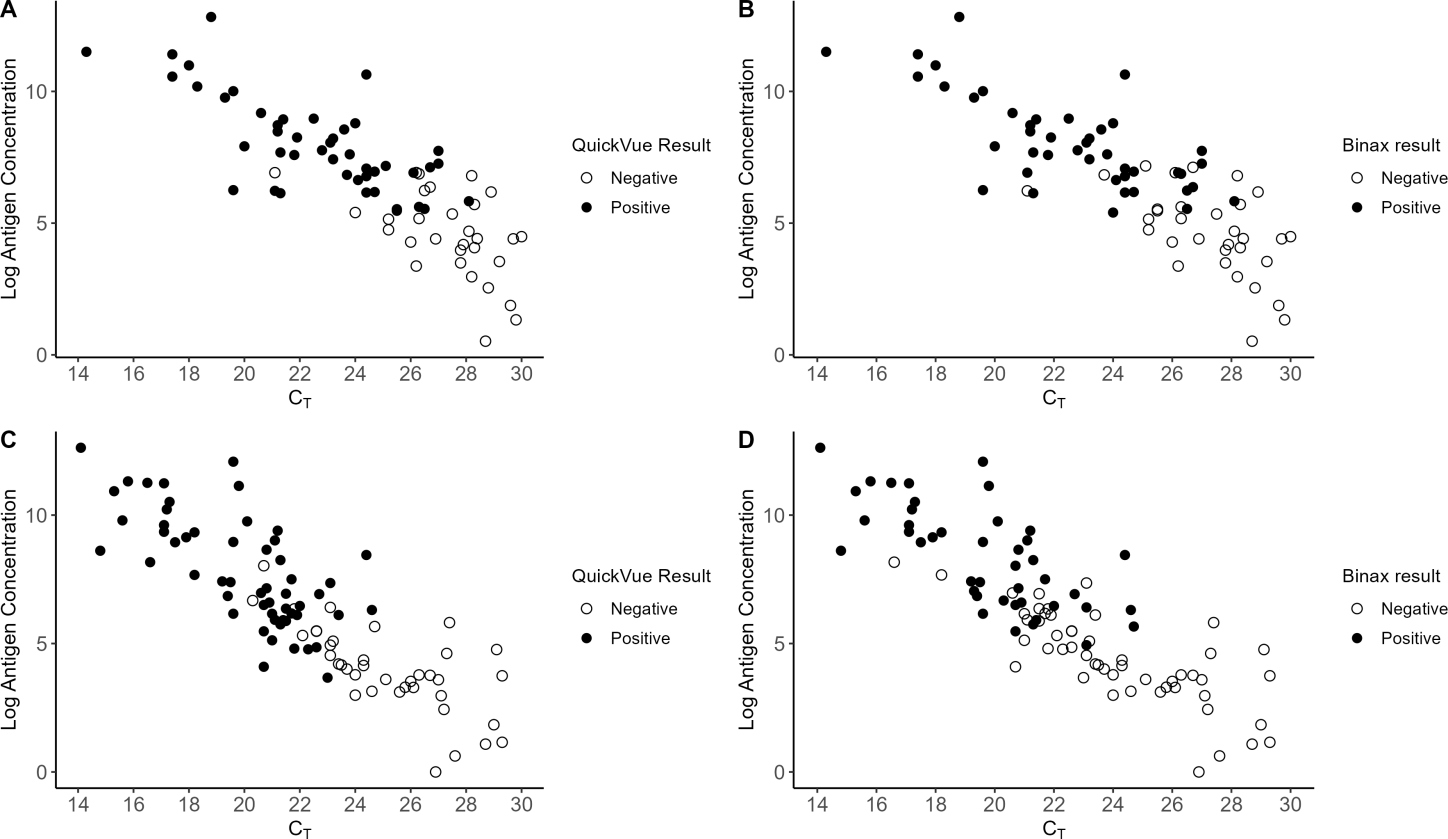
Results of testing two commercially available rapid antigen tests against individual remnant clinical samples for Delta (**A and B**) and Omicron (**C and D**). Sequence-verified residual mid turbinate samples from 75 individuals with Delta infection and 84 individuals with BA.1 infection underwent RT-PCR testing using the Xpert Xpress CoV-2/Flu/RSV plus & Xpert Xpress SARS-CoV-2 assays (Cepheid), protein quantification using the Simoa SARS-CoV-2 N Protein Antigen assay (Quanterix), and rapid antigen testing (QuickVue or Binax) according to the manufacturer’s instructions. *Y*-axes reflect natural log(*n* + 1) transformed antigen concentration. Abbreviations: Cycle threshold (C_T_).

From this study population, 75 Delta and 84 Omicron samples with *C*_*T*_ less than 30 (Cepheid Xpert assay; described in supplementary methods) were randomly selected for testing with the Abbott BinaxNOW COVID-19 Antigen Test and Quidel QuickVue SARS Antigen Test RATs. Across all samples, sensitivity was similar between Delta and Omicron for Quickvue. Sensitivity appeared lower for Omicron than Delta samples for BinaxNow, although this difference was not statistically significant (Table 2). As expected, tests were more sensitive in samples with higher concentrations of viral antigen and lower *C*_*T*_ values (Fig. 2A through D).

**TABLE 2:** Sensitivity of BinaxNOW and QuickVue RATs using individual RCS^*a*^

	BinaxNow			QuickVue		
	Delta	Omicron	P-value	Delta	Omicron	P-value
Overall	57.3 (56.2, 58.4)	45.7 (44.7, 46.8)	0.18	60 (58.9, 61.1)	58.5 (57.5, 59.5)	0.97
Antigen Concentration (pg/mL)						
≥10	59.7 (48.4, 71)	48.3 (37.9, 58.7)	0.20	62.5 (51.3, 73.7)	62.5 (52.4, 72.6)	1
≥100	72.88 (61.5, 84.2)	65.15 (53.7, 76.6)	0.46	76.3 (65.4, 87.1)	81.5 (72.1, 91)	0.51
≥1000	91.2 (81.6, 100.7)	85.7 (74.1, 97.3)	0.74	94.1 (86.2, 100)	97.1 (91.4, 100)	1
≥10000	100 (--, --)	100 (--, --)	--	100 (--, --)	100 (--, --)	--
C_T_ Value						
≤20	100 (100, 100)	91.7 (90.6, 100)	0.89	100 (100, 100)	95.8 (95.0, 100)	1
≤22	95 (94.0, 100)	72.6 (71.3, 73.8)	0.08	95 (94.0, 100)	92.2 (91.4, 100)	1
≤24	93.3 (92.4, 100)	58.0 (56.8, 59.1)	0.001	93.3 (92.4, 100)	76.8 (75.8, 77.8)	0.09
≤26	81.4 (80.2, 82.6)	54.4 (53.3, 55.5)	0.01	88.4 (87.4, 100)	69.6 (68.6, 70.6)	0.03
≤28	70 (68.8, 71.2)	48.3 (47.3, 49.4)	0.01	73.3 (72.2, 74.4)	61.8 (60.8, 62.8)	0.16

^*a*^ Samples were stratified by antigen concentration (top panel) or *C*_*T*_ value (bottom panel). Antigen concentration was measured using the Simoa SARS-CoV-2 N Protein Antigen assay (Quanterix), and *C*_*T*_ was measured using the Cepheid GeneXpert Dx Instrument System with Xpert Xpress CoV-2/Flu/RSV plus cartridges (EUA 302-6991, Rev. B., October 2021). The sensitivity of detection with corresponding 95% confidence intervals for Delta and Omicron was compared within each stratum using chi-square or Fisher’s exact test. The supplementary data file contains results of BinaxNOW and QuickVue for each observation.

When samples were stratified by antigen concentration (Table 2, top panel), QuickVue had similar sensitivity for Delta and Omicron across all strata. BinaxNow appeared somewhat less sensitive for Omicron than Delta, but this difference was not statistically significant. For both assays and both variants, sensitivity increased as antigen concentration increased, as expected. When samples were stratified by *C*_*T*_ value, sensitivity decreased as *C*_*T*_ value increased, as expected (Table 2, bottom panel). Both assays showed lower sensitivity for Omicron than Delta for *C*_*T*_ thresholds 24 and higher; this result was more pronounced, and only statistically significant, for BinaxNOW.

Thus, results from both individual RCS and RCS pools show that the QuickVue assay has similar sensitivity in detecting Delta and Omicron when antigen concentration is used as a comparator; it has a somewhat (but not statistically significant) reduced sensitivity for Omicron when using *C*_*T*_ value as a comparator. The BinaxNOW assay has a somewhat (but not statistically significant) reduced sensitivity for Omicron when antigen concentration is used as a comparator; it has a more pronounced (and statistically significant) reduction in sensitivity for Omicron when *C*_*T*_ value is used as a comparator. Results for both individual RCS and RCS pools showed a discrepancy in RAT sensitivity when using antigen concentration versus *C*_*T*_ value as the comparator.

To identify potential mutations that might affect test performance, we analyzed SARS-CoV-2 genome sequences. The Delta samples represented a range of sublineages (Supplementary Data File). In the N protein, aside from the four lineage-defining mutations (D63G, R203M, G215C, and D377Y), no mutation was present in more than three samples. The Omicron samples all belonged to lineage BA.1 or BA.1.1. In addition to lineage-defining mutations (P13L, R203K, G204R, and DEL31-33), 25 of the 152 samples had D343G and 4 had P67S. The Omicron lineages that have emerged since the time of this study, BA.2, BA.4, and BA.5, contain the additional mutation S413R, which was not present in any of these samples. Overall, sequence analysis confirmed that these clinical samples were representative of Delta and Omicron variants and suggested that, if N protein mutations affect test sensitivity, they are likely to be lineage-defining mutations.

### Omicron samples have lower antigen-per-RNA than Delta samples

**Fig 3:**
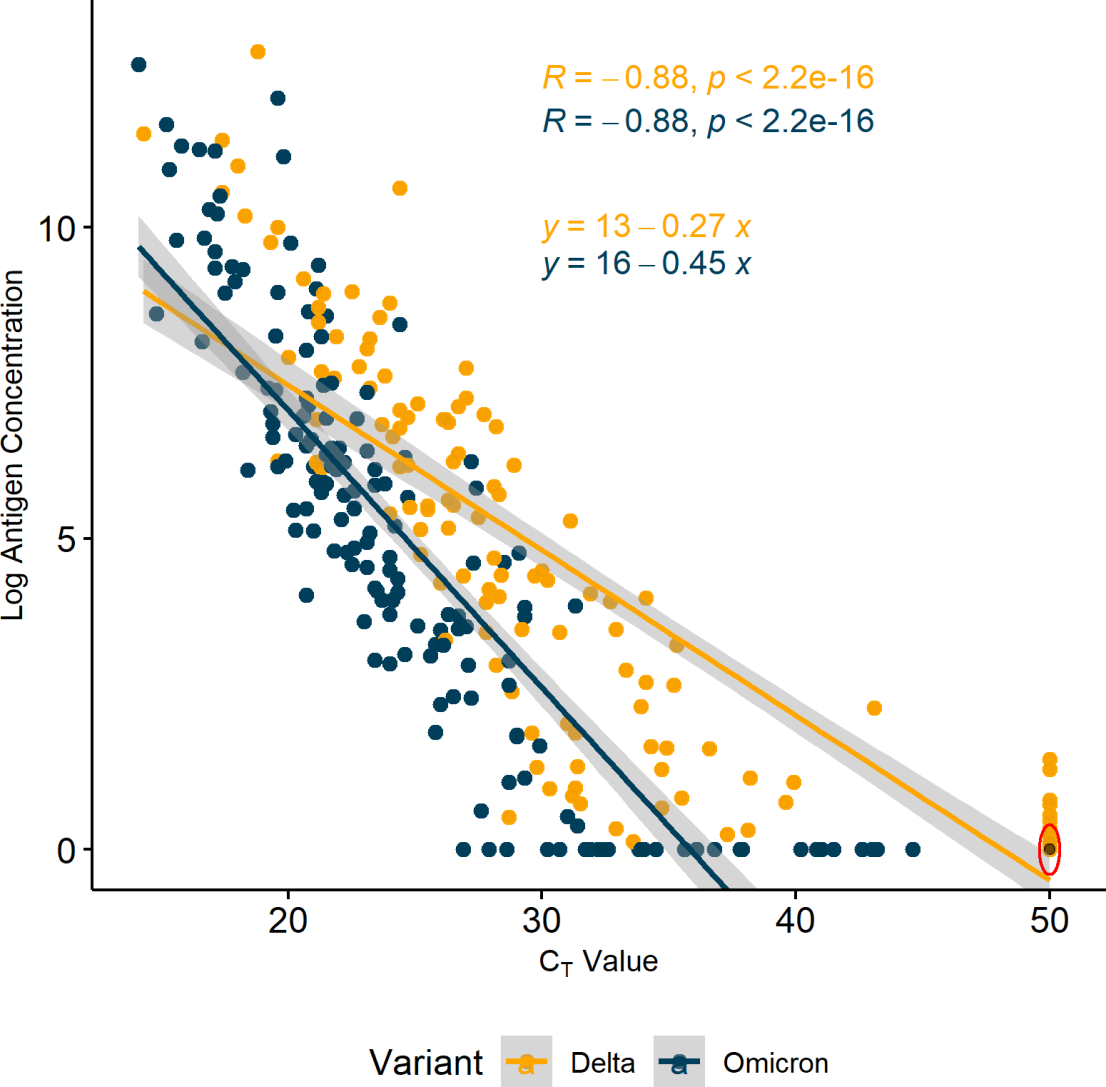
Correlation between antigen concentration and *C*_*T*_ value for individual remnant clinical samples. Sequence-verified residual mid turbinate samples from 163 individuals with Delta infection and 169 individuals with BA.1 infection underwent RT-PCR testing using the Xpert Xpress CoV-2/Flu/RSV plus & Xpert Xpress SARS-CoV-2 assays (Cepheid) and protein testing using the Simoa SARS-CoV-2 N Protein Antigen assay (Quanterix), according to the manufacturer’s instructions. *Y*-axes reflect natural log(*n* + 1) transformed antigen concentration. The red circle signifies 41 Delta samples whose *C*_*T*_ values were above the assay detection limit and antigen concentrations were below the assay detection limit. Abbreviations: Cycle threshold (*C*_*T*_).

We formally compared the relationship between antigen concentration and *C*_*T*_ in 163 Delta and 169 Omicron individual RCS, including the samples tested by RAT. As expected, antigen concentration and *C*_*T*_ were highly correlated, both across all samples and for each variant individually (Fig. 3), including in sensitivity analysis (Fig. S4). However, the slope of the correlation line was different for Omicron and Delta, suggesting a different relationship between antigen and *C*_*T*_ for each variant. Notably, regression analysis indicated a significant association between *C*_*T*_ value and variant, for a given antigen concentration (Table 3). Specifically, Omicron samples had a 6.8 [standard error (SE)=0.55] cycle lower *C*_*T*_ than Delta samples, for a given antigen concentration (*P* value < 0.001), indicating a greater amount of RNA-per-antigen (a lower amount of antigen-per-RNA) than Delta samples. In the full regression model that also included vaccine status, presence of symptoms, and duration of symptoms, Omicron samples had a 7.7 (SE = 0.70, *P* value < 0.001) cycle lower *C*_*T*_ than Delta samples, for a given antigen concentration. Unsurprisingly, *C*_*T*_ value was significantly associated with antigen concentration, with a decrease of 2.3 (SE = 0.08, *P* value < 0.001) cycles and 2.2 (SE = 0.08, *P* value < 0.001) cycles per natural log change in antigen concentration in the base model and full model, respectively. *C*_*T*_ value was also significantly associated with the presence of symptoms; it was 6.6 (SE = 2.23, *P* value = 0.003) cycles lower for individuals with 3 days or less of symptoms, 8.7 (SE = 2.20, *P* value < 0.001) cycles lower for individuals with a symptom duration between 4 and 7 days, and 8.1 (SE = 2.28, *P* value < 0.001) cycles lower for individuals with symptoms lasting longer than 7 days compared to asymptomatic individuals. We did not observe a significant association between *C*_*T*_ value and vaccine status (Beta = 0.37, SE = 1.34, *P* value = 0.80). In sensitivity analysis, these relationships remained similar and statistically significant, but with lower magnitude of effect (Table S1).

**TABLE 3:** Association between *C*_*T*_ value and natural log(*n* + 1) transformed antigen concentration (pg/mL), variant, vaccine status, and presence and duration of symptoms for 151 Delta and 168 Omicron samples, excluding samples with missing information on days since last vaccine or symptom duration

	Base model			Full model		
Variable	Beta^*b*^	SE^*a*^	*P* value	Beta	SE	*P* value
Log (antigen concentration +1)	−2.3	0.08	<0.001	−2.2	0.08	<0.001
Variant						
Delta	Ref	—		Ref	—	
Omicron	−6.8	0.55	<0.001	−7.7	0.70	<0.001
Vaccine status						
Not fully vaccinated				Ref	—	
Fully vaccinated				0.37	1.34	0.8
Presence of symptoms						
Asymptomatic				Ref	—	
Symptomatic ≤3 days				−6.6	2.23	0.003
Symptomatic 4–7 days				−8.7	2.20	<0.001
Symptomatic >7 days				−8.1	2.28	<0.001
Days since last vaccine						
Unvaccinated				Ref	—	
Within the last 90 days				0.51	1.30	0.7
Between 91 and 180 days ago				0.23	1.53	0.9
Between 181 and 270 days ago				−0.27	1.49	0.9
More than 270 days ago				1.5	1.86	0.4

^*a*^ SE = Standard Error, Ref = Reference Level.

^*b*^ Beta coefficients have been rounded but percentage change calculations were computed before rounding and therefore may be different.

The magnitude of *C*_*T*_ difference between Delta and Omicron samples [7.7 cycles (SE = 0.70), for a given antigen concentration] was greater than the mean *C*_*T*_ difference expected from the different number of freeze-thaws they had undergone [2.1 cycles (SE = 0.32), with a concomitant decrease in antigen concentration by 16%, Supplementary Material; Fig. S5; Table S2]. Thus, we infer that differential freeze-thaw conditions are likely to explain some, but not all, of the discrepancy we observed. The observation that Omicron samples have a lower amount of antigen-per-RNA also helps to explain our finding that rapid antigen test sensitivity is different when using *C*_*T*_ versus antigen concentration as a comparator for RCS pools, all of which underwent the same number of freeze-thaws.

### Omicron samples have lower infectivity than Delta samples

Given the observed discrepancy between protein concentration and *C*_*T*_ value for Delta and Omicron RCS in this study, we assessed whether there was a difference in virus infectivity from these samples. Seventy-five Delta and 85 Omicron clinical samples with *C*_*T*_ <30 were tested against Calu-3 cells in duplicate. Calu-3 cells were infected by 37 (49.3%) of Delta and 37 (43.5%) of Omicron samples (Fig. S6). ELISpot panels showing representative data are shown in Fig. S7. Interestingly, infectivity appeared to be inversely associated with *C*_*T*_ value for Delta but not Omicron samples (Fig. S6). We formally assessed this using logistic regression analysis. In univariate analysis, Calu-3 infectivity was inversely associated with *C*_*T*_ value for Delta [odds ratio (OR) = 0.75, 95% confidence interval (CI): 0.63–0.88] but not Omicron (OR = 1.03, 95% CI: 0.90–1.17), and was not associated with antigen concentration, vaccine status, symptom duration, or age (Table S3). In multivariate analysis, Calu-3 infectivity remained inversely associated with *C*_*T*_ value for Delta (Table S3). Specifically, for every one-cycle increase in *C*_*T*_ value, the odds of having a positive Calu-3 result decreased by 28% (95% CI: 14–42%) for Delta samples. Again, there was no association between Calu-3 infectivity and antigen concentration for either Delta or Omicron samples.

Thus, Omicron samples in this study had less antigen-per-RNA and less infectivity than Delta samples, and there was no association between RNA level (as measured by *C*_*T*_) and infectivity for Omicron samples.

## DISCUSSION

Overall, we found that most commercially available RATs had similar sensitivity in detecting Omicron and Delta when antigen concentration was used as a comparator. However, when *C*_*T*_ value was used as a comparator, most RATs had a lower sensitivity for Omicron than Delta.

These findings are largely consistent with prior studies showing lower sensitivity of RATs in detecting Omicron than Delta when using *C*_*T*_ value as a comparator, especially for samples with low RNA concentration. Osterman et al. found a 10- to 100-fold higher LoD for Omicron compared to Delta among nine RATs in Germany (these tests did not overlap with the tests used in our study) (2). Bayart et al. found lower sensitivity for Omicron (0–23%) than Delta (32–80%) across six RATs in Belgium for clinical samples with *C*_*T*_ >25 (14). Bekliz et al. found lower sensitivity for Omicron than Delta across seven RATs (four of which were statistically significant), and Landaverde et al. found low sensitivity of BinaxNOW for detecting Omicron especially with *C*_*T*_ >23 (15, 16). Few studies have shown a high sensitivity of BinaxNOW for Omicron (4, 5). A few other studies reported similar sensitivity for RATs in detecting Omicron and Delta, but these were based on serially diluted, cultured virus, not clinical samples. For example, Deerain et al. reported high sensitivity for both variants up to *C*_*T*_ = 25 and essentially no detection at *C*_*T*_ = 28 for 10 RATs (mostly non-overlapping with ours) (17). Stanley et al. found decreased sensitivity for Delta compared to Omicron and WA1 (18). All these prior studies used *C*_*T*_ value or RNA concentration as a comparator, and none reported antigen concentration. Thus overall, there is accumulating evidence that many RATs demonstrate lower sensitivity for Omicron than Delta for primary clinical samples when using *C*_*T*_ value as a comparator, consistent with our findings.

Our study using clinical samples offers a potential explanation for the apparent lower sensitivity of RATs for Omicron, by investigating variant-specific discrepancies in antigen concentration versus *C*_*T*_ value. Specifically, the Omicron samples in this study had a lower amount of antigen-per-RNA than the Delta samples, and because RATs detect antigen rather than RNA, they appear less sensitive for Omicron when *C*_*T*_ value is used as a comparator. By contrast, when we used antigen concentration as a comparator (an “apples to apples” comparison), we found that most RATs had similar sensitivity for Omicron and Delta.

We considered several technical factors that could account for differences in measurement of RNA and antigen concentration between variants in this study. Whereas Delta clinical samples underwent RNA and protein concentration testing after two freeze-thaw cycles, Omicron clinical samples were tested fresh. Results from our freeze-thaw experiment suggest that this difference in sample handling could account for some, but not all, of the observed variant-specific differences in the ratio between RNA (*C*_*T*_ value) and antigen concentration. Specifically, our freeze-thaw experiment demonstrated a difference of about 2 *C*_*T*_ after two freeze-thaws, whereas the difference between Omicron and Delta was over 7 *C*_*T*_. Additionally, RCS pools, which had undergone the same handling conditions for both Delta and Omicron (i.e., samples in both variant pools had the same number of freeze-thaws), also showed a discrepancy in results when antigen versus RNA concentration was used as a comparator. Because our results were consistent across two different RT-PCR assays [Xpert Xpress CoV-2/Flu/RSV *plus* assay (Cepheid) for individual RCS and CDC N2 assay for RCS pools], differences in RT-PCR efficiency are unlikely to account for our findings.

We also considered whether Omicron-specific mutations may have affected the performance of diagnostic antibodies used in these assays. A recent study mapped the N protein epitopes recognized by antibodies in many SARS-CoV-2 antigen tests and identified escape mutations using deep mutational scanning (19). The key amino acid positions identified considering both antibodies used by the Simoa SARS-CoV-2 N Protein Antigen Test (Quanterix) (5, 36–41, 51–53, 56, 62, 66, 71–73, 82–87, 95, 98–101, 108–117, 128–133, 143, 158–161, 167, and 171–173) are not canonically mutated in Omicron, and were not specifically mutated in the samples from this study. The same study assessed antibodies for several RATs included here and found no overlap between variant-specific mutations and antibody escape mutations. Thus, variant-specific mutations are unlikely to account for differences in the measurement of antigen concentration in this study.

Overall, we infer that the Omicron samples in this study truly had a lower amount of antigen-per-RNA than the Delta samples. There are several potential explanations for this based on viral dynamics over the course of infection. Early studies of SARS-CoV-2 showed that viral load generally peaks around day 3 of viral shedding, just before or at the time of symptom onset, and clears after 7–10 days (20 – 23). Antigen detection peaks later, generally several days after symptom onset (24), and thus antigen detection frequently lags behind RNA detection (25). Individuals in our study infected with Omicron presented for testing sooner after symptom onset than individuals infected with Delta, and thus our Omicron samples may have been collected at a time when antigen levels may still have lagged behind RNA levels.

In addition, there may be variant-specific differences in viral dynamics, because of either intrinsic biological differences in viral replication and pathogenesis, and/or differences in the characteristics of individuals who are infected with each variant. For example, due to the timing of vaccine booster rollouts, individuals infected with Omicron are likely to have been vaccinated more recently (and with more doses) than individuals infected with Delta. Consistent with this, individuals in our study infected with Omicron had been vaccinated more recently than individuals infected with Delta. In a recent study, boosted individuals infected with Omicron were slower to clear viral RNA than unboosted individuals infected with Delta or Omicron, but the effect on antigen dynamics remains unknown (26). In another study, individuals with Omicron infection had higher *C*_*T*_ values than individuals with pre-Omicron variants, controlling for vaccine status and symptom duration, though antigen concentration was not measured (6). Thus, there may also be variant-specific differences in the viral lifecycle that lead to differences in RNA and antigen concentration, such as differential sgRNA transcription, gene expression, protein degradation, or protein aggregation. Compatible with our findings, a recent report demonstrated a decrease in the sensitivity of RATs over time since the start of the pandemic, including during the Omicron era (27). The authors posited that increased immunity, including through vaccination, led to early symptom onset and early testing, before epithelial cell shedding had generated high concentrations of nucleocapsid protein.

Together with these prior studies, our results support a model in which individuals infected with Omicron presented for testing earlier in the course of infection, when antigen concentration lagged behind RNA concentration, leading to an apparent decrease in rapid antigen test sensitivity when *C*_*T*_ value is used as a comparator. There are likely multiple factors contributing to the earlier presentation for testing of individuals infected with Omicron, one of which may be a more rapid and robust symptom onset due to recent/boosted vaccination.

Interestingly, we also found that the Omicron samples in this study had lower infectivity than the Delta samples and there was no correlation between *C*_*T*_ value and infectivity for Omicron samples. These findings are compatible with, and are likely explained by, recent observations that Omicron cell entry has a greater dependence on receptor-mediated endocytosis than TMPRSS2-mediated spike cleavage and fusion (28) and Omicron replicates less well in TMPRSS2 expressing cells (29 – 31).

This study has several limitations. First, due to the necessary logistical constraints of comparing contemporary to banked samples, the individual RCS tested in this study had undergone different handling for Omicron versus Delta samples. This was mitigated to some extent by also testing pooled RCS, and by explicitly testing the effects of freeze-thaw cycles. In addition, while we tested ten commercially available RATs using pooled RCS, we were only able to test individual RCS against two RATs, given constraints in sample volume. Finally, the marked differences we observed in infectivity between Omicron and Delta samples must be interpreted in light of recent studies showing important variant-specific differences in cell entry and cell biology.

Nevertheless, our results have important implications for clinical practice and public health. First, we show that the choice of comparator assay plays an important role in interpreting the results of sensitivity evaluations for RATs. Future studies will benefit from the use of well-characterized and standardized reference materials to use in assay testing, as well as careful consideration of duration of symptoms at the time of sample collection. Interestingly, based on our findings, the BinaxNOW assay seems to be the most adversely affected by the Omicron variant relative to its performance against Delta variant, which has practical public health implications given its wide use and large market share in the United States. By contrast, most commercially available RATs have similar sensitivity for detecting Omicron and Delta, when antigen concentration is used as a comparator. This reinforces the effectiveness of existing tests, while also emphasizing the point that a negative RDT early in SARS-CoV-2 infection may have low negative predictive value, and RDT testing should be repeated over time. However, within-patient viral dynamics are evolving throughout the pandemic, likely due to changes in both the virus and the host (e.g., vaccination). Further work is needed to investigate the causes and mechanisms of variant-specific differences in RNA concentration, antigen concentration, infectivity, and viral dynamics, particularly as new variants continue to emerge.

## Data Availability

All new SARS-CoV-2 sequences generated for this study are available in NCBI under BioProject PRJNA634356 with GenBank accession numbers OP453746-OP453966 and OP654747-OP654750. Individual GenBank accession numbers and GISAID accession numbers for each sample are listed in the Supplementary Data File.

## SUPPLEMENTAL MATERIAL

Supplementary Material jcm.00138-23-s0001.docx Supplementary methods, results, figures, and tables

Supplemental Data File 1: Remnant clinical sample information jcm.00138-23-s0002.csv Full demographic, clinical, and testing results for the 334 remnant clinical samples used in this study

ASM does not own the copyrights to Supplemental Material that may be linked to, or accessed through, an article. The authors have granted ASM a non-exclusive, world-wide license to publish the Supplemental Material files. Please contact the corresponding author directly for reuse.

## References

[1] U.S. Food and Drug Administration . 2019. In vitro diagnostics EUAs - antigen diagnostic tests for SARS-CoV-2. Available from: https://www.fda.gov/medical-devices/coronavirus-disease-2019-covid-19-emergency-use-authorizations-medical-devices/in-vitro-diagnostics-euas-antigen-diagnostic-tests-sars-cov-2#iaft1

[2] Osterman A , Badell I , Basara E , Stern M , Kriesel F , Eletreby M , Öztan GN , Huber M , Autenrieth H , Knabe R , Späth PM , Muenchhoff M , Graf A , Krebs S , Blum H , Durner J , Czibere L , Dächert C , Kaderali L , Baldauf H-M , Keppler OT . 2022. Impaired detection of Omicron by SARS-CoV-2 rapid antigen tests. Med Microbiol Immunol 211:105–117. doi:10.1007/s00430-022-00730-z 35187580 PMC8858605

[3] Schuit E , Venekamp RP , Veldhuijzen IK , van den Bijllaardt W , Pas SD , Stohr J , Lodder EB , Hellwich M , Molenkamp R , Igloi Z , Wijers C , Vroom IH , Nagel-Imming CRS , Han WGH , Kluytmans J , van den Hof S , van de Wijgert J , Moons KGM . 2022. Head-to-head comparison of the accuracy of saliva and nasal rapid antigen SARS-CoV-2 self-testing: cross-sectional study. BMC Med 20:406. doi:10.1186/s12916-022-02603-x 36280827 PMC9590385

[4] Schrom J , Marquez C , Petersen M , DeRisi J , Havlir D . 2022. Comparison of SARS-CoV-2 reverse transcriptase polymerase chain reaction and BinaxNOW rapid antigen tests at a community site during an Omicron surge. Ann Intern Med 175:W119. doi:10.7326/L22-0257 36252254

[5] Eyre DW , Futschik M , Tunkel S , Wei J , Cole-Hamilton J , Saquib R , Germanacos N , Dodgson AR , Klapper PE , Sudhanva M , Kenny C , Marks P , Blandford E , Hopkins S , Peto TEA , Fowler T . 2023. Performance of antigen lateral flow devices in the UK during the alpha, Delta, and Omicron waves of the SARS-CoV-2 pandemic: a diagnostic and observational study. Lancet Infect Dis 23:922–932. doi:10.1016/S1473-3099(23)00129-9 37001541 PMC10048397

[6] Tassetto M , Garcia-Knight M , Anglin K , Lu S , Zhang A , Romero M , Pineda-Ramirez J , Sanchez RD , Donohue KC , Pfister K , Chan C , Saydah S , Briggs-Hagen M , Peluso MJ , Martin JN , Andino R , Midgley CM , Kelly JD . 2022. Detection of higher cycle threshold values in culturable SARS-CoV-2 Omicron BA.1 sublineage compared with pre-Omicron variant specimens - San Francisco bay area, California, July 2021-March 2022. MMWR Morb Mortal Wkly Rep 71:1151–1154. doi:10.15585/mmwr.mm7136a3 36074732 PMC9470222

[7] Walker AS , Pritchard E , House T , Robotham JV , Birrell PJ , Bell I , Bell JI , Newton JN , Farrar J , Diamond I , Studley R , Hay J , Vihta K-D , Peto TE , Stoesser N , Matthews PC , Eyre DW , Pouwels KB , COVID-19 Infection Survey team . 2021. Ct threshold values, a proxy for viral load in community SARS-CoV-2 cases, demonstrate wide variation across populations and over time. Elife 10:e64683. doi:10.7554/eLife.64683 34250907 PMC8282332

[8] Iannone R , Cheng J , Schloerke B , Hughes E . 2022. Gt: Easily create presentation-ready display tables. R package version 0.7.0. Ed2022

[9] Sjoberg DD , Whiting K , Curry M , Lavery JA , Larmarange J . 2021. Reproducible summary tables with the gtsummary package. R J 13:570. doi:10.32614/RJ-2021-053

[10] Kassambara A . ggpubr: 'ggplot2' based publication ready plots. R package version 0.4.0 ed2020

[11] Hadley W . stringr: simple, consistent wrappers for common string operations. R package version 1.4.1 ed2022

[12] Wickham H . 2016. ggplot2: elegant graphics for data analysis. Springer-Verlag, New York.

[13] Shan D , Johnson JM , Fernandes SC , Suib H , Hwang S , Wuelfing D , Mendes M , Holdridge M , Burke EM , Beauregard K , Zhang Y , Cleary M , Xu S , Yao X , Patel PP , Plavina T , Wilson DH , Chang L , Kaiser KM , Nattermann J , Schmidt SV , Latz E , Hrusovsky K , Mattoon D , Ball AJ . 2021. N-protein presents early in blood, dried blood and saliva during asymptomatic and symptomatic SARS-CoV-2 infection. Nat Commun 12:1931. doi:10.1038/s41467-021-22072-9 33771993 PMC7997897

[14] Bayart J-L , Degosserie J , Favresse J , Gillot C , Didembourg M , Djokoto HP , Verbelen V , Roussel G , Maschietto C , Mullier F , Dogné J-M , Douxfils J . 2022. Analytical sensitivity of six SARS-CoV-2 rapid antigen tests for Omicron versus Delta variant. Viruses 14:654. doi:10.3390/v14040654 35458384 PMC9031584

[15] Bekliz M , Adea K , Puhach O , Perez-Rodriguez F , Marques Melancia S , Baggio S , Corvaglia A-R , Jacquerioz F , Alvarez C , Essaidi-Laziosi M , Escadafal C , Kaiser L , Eckerle I . 2022. Analytical sensitivity of eight different SARS-CoV-2 antigen-detecting rapid tests for Omicron-BA.1 variant. Microbiol Spectr 10:e0085322. doi:10.1128/spectrum.00853-22 35938792 PMC9430749

[16] Landaverde L , Turcinovic J , Doucette-Stamm L , Gonzales K , Platt J , Connor JH , Klapperich C . 2022. Comparison of BinaxNOW and SARS-CoV-2 qRT-PCR detection of the Omicron variant from matched anterior nares swabs. Microbiol Spectr 10:e0130722. doi:10.1128/spectrum.01307-22 36255297 PMC9769721

[17] Deerain J , Druce J , Tran T , Batty M , Yoga Y , Fennell M , Dwyer DE , Kok J , Williamson DA . 2022. Assessment of the analytical sensitivity of 10 lateral flow devices against the SARS-CoV-2 Omicron variant. J Clin Microbiol 60:e0247921. doi:10.1128/jcm.02479-21 34936477 PMC8849215

[18] Stanley S , Hamel DJ , Wolf ID , Riedel S , Dutta S , Contreras E , Callahan CJ , Cheng A , Arnaout R , Kirby JE , Kanki PJ . 2022. Limit of detection for rapid antigen testing of the SARS-CoV-2 Omicron and Delta variants of concern using live-virus culture. J Clin Microbiol 60:e0014022. doi:10.1128/jcm.00140-22 35440165 PMC9116160

[19] Frank F , Keen MM , Rao A , Bassit L , Liu X , Bowers HB , Patel AB , Cato ML , Sullivan JA , Greenleaf M , Piantadosi A , Lam WA , Hudson WH , Ortlund EA . 2022. Deep mutational scanning identifies SARS-CoV-2 nucleocapsid escape mutations of currently available rapid antigen tests. Cell 185:3603–3616. doi:10.1016/j.cell.2022.08.010 36084631 PMC9420710

[20] Stankiewicz Karita HC , Dong TQ , Johnston C , Neuzil KM , Paasche-Orlow MK , Kissinger PJ , Bershteyn A , Thorpe LE , Deming M , Kottkamp A , Laufer M , Landovitz RJ , Luk A , Hoffman R , Roychoudhury P , Magaret CA , Greninger AL , Huang M-L , Jerome KR , Wener M , Celum C , Chu HY , Baeten JM , Wald A , Barnabas RV , Brown ER . 2022. Trajectory of viral RNA load among persons with incident SARS-CoV-2 G614 infection (Wuhan strain) in association with COVID-19 symptom onset and severity. JAMA Netw Open 5:e2142796. doi:10.1001/jamanetworkopen.2021.42796 35006245 PMC8749477

[21] Kissler SM , Fauver JR , Mack C , Olesen SW , Tai C , Shiue KY , Kalinich CC , Jednak S , Ott IM , Vogels CBF , Wohlgemuth J , Weisberger J , DiFiori J , Anderson DJ , Mancell J , Ho DD , Grubaugh ND , Grad YH , Riley S . 2021. Viral dynamics of acute SARS-CoV-2 infection and applications to diagnostic and public health strategies. PLoS Biol 19:e3001333. doi:10.1371/journal.pbio.3001333 34252080 PMC8297933

[22] Cevik M , Tate M , Lloyd O , Maraolo AE , Schafers J , Ho A . 2021. SARS-CoV-2, SARS-CoV, and MERS-CoV viral load dynamics, duration of viral shedding, and Infectiousness: a systematic review and meta-analysis. Lancet Microbe 2:e13–e22. doi:10.1016/S2666-5247(20)30172-5 33521734 PMC7837230

[23] Jones TC , Biele G , Mühlemann B , Veith T , Schneider J , Beheim-Schwarzbach J , Bleicker T , Tesch J , Schmidt ML , Sander LE , Kurth F , Menzel P , Schwarzer R , Zuchowski M , Hofmann J , Krumbholz A , Stein A , Edelmann A , Corman VM , Drosten C . 2021. Estimating infectiousness throughout SARS-CoV-2 infection course. Science 373:eabi5273. doi:10.1126/science.abi5273 34035154 PMC9267347

[24] Chu VT , Schwartz NG , Donnelly MAP , Chuey MR , Soto R , Yousaf AR , Schmitt-Matzen EN , Sleweon S , Ruffin J , Thornburg N , Harcourt JL , Tamin A , Kim G , Folster JM , Hughes LJ , Tong S , Stringer G , Albanese BA , Totten SE , Hudziec MM , Matzinger SR , Dietrich EA , Sheldon SW , Stous S , McDonald EC , Austin B , Beatty ME , Staples JE , Killerby ME , Hsu CH , Tate JE , Kirking HL , Matanock A , COVID-19 Household Transmission Team . 2022. Comparison of home antigen testing with RT-PCR and viral culture during the course of SARS-CoV-2 infection. JAMA Intern Med 182:701–709. doi:10.1001/jamainternmed.2022.1827 35486394 PMC9055515

[25] Soni A , Herbert C , Filippaios A , Broach J , Colubri A , Fahey N , Woods K , Nanavati J , Wright C , Orwig T , Gilliam K , Kheterpal V , Suvarna T , Nowak C , Schrader S , Lin H , O’Connor L , Pretz C , Ayturk D , Orvek E , Flahive J , Lazar P , Shi Q , Achenbach C , Murphy R , Robinson M , Gibson L , Stamegna P , Hafer N , Luzuriaga K , Barton B , Heetderks W , Manabe YC , McManus D . 2022. Comparison of rapid antigen tests' performance between Delta and Omicron variants of SARS-CoV-2: a secondary analysis from a serial home self-testing study. Ann Intern Med 175:1685–1692. doi:10.7326/M22-0760 36215709 PMC9578286

[26] Hay JA , Kissler SM , Fauver JR , Mack C , Tai CG , Samant RM , Connolly S , Anderson DJ , Khullar G , MacKay M , Patel M , Kelly S , Manhertz A , Eiter I , Salgado D , Baker T , Howard B , Dudley JT , Mason CE , Nair M , Huang Y , DiFiori J , Ho DD , Grubaugh ND , Grad YH . 2022. Quantifying the impact of immune history and variant on SARS-CoV-2 viral kinetics and infection rebound: a retrospective cohort study. Elife 11. doi:10.7554/eLife.81849 PMC9711520 36383192

[27] Meiners L , Horn J , Mühlemann B , Schmidt ML , Walper F , Menzel P , Schwarzer R , Rose R , Krumbholz A , Jones TC , Corman VM , Seybold J , Drosten C . 2020. SARS-CoV-2 rapid antigen test sensitivity and viral load in freshly symptomatic hospital employees, December 2020 to February 2022. SSRN Journal. doi:10.2139/ssrn.4099425 38759669

[28] Willett BJ , Grove J , MacLean OA , Wilkie C , De Lorenzo G , Furnon W , Cantoni D , Scott S , Logan N , Ashraf S , Manali M , Szemiel A , Cowton V , Vink E , Harvey WT , Davis C , Asamaphan P , Smollett K , Tong L , Orton R , Hughes J , Holland P , Silva V , Pascall DJ , Puxty K , da Silva Filipe A , Yebra G , Shaaban S , Holden MTG , Pinto RM , Gunson R , Templeton K , Murcia PR , Patel AH , Klenerman P , Dunachie S , PITCH Consortium, COVID-19 Genomics UK (COG-UK) Consortium, Haughney J , Robertson DL , Palmarini M , Ray S , Thomson EC . 2022. SARS-CoV-2 Omicron is an immune escape variant with an altered cell entry pathway. Nat Microbiol 7:1709. doi:10.1038/s41564-022-01241-6 36114232 PMC9483304

[29] Meng B , Abdullahi A , Ferreira IATM , Goonawardane N , Saito A , Kimura I , Yamasoba D , Gerber PP , Fatihi S , Rathore S , Zepeda SK , Papa G , Kemp SA , Ikeda T , Toyoda M , Tan TS , Kuramochi J , Mitsunaga S , Ueno T , Shirakawa K , Takaori-Kondo A , Brevini T , Mallery DL , Charles OJ , CITIID-NIHR BioResource COVID-19 Collaboration, Genotype to Phenotype Japan (G2P-Japan) Consortium, Ecuador-COVID19 Consortium, Bowen JE , Joshi A , Walls AC , Jackson L , Martin D , Smith KGC , Bradley J , Briggs JAG , Choi J , Madissoon E , Meyer KB , Mlcochova P , Ceron-Gutierrez L , Doffinger R , Teichmann SA , Fisher AJ , Pizzuto MS , de Marco A , Corti D , Hosmillo M , Lee JH , James LC , Thukral L , Veesler D , Sigal A , Sampaziotis F , Goodfellow IG , Matheson NJ , Sato K , Gupta RK . 2022. Altered TMPRSS2 usage by SARS-CoV-2 Omicron impacts infectivity and fusogenicity. Nature 603:706–714. doi:10.1038/s41586-022-04474-x 35104837 PMC8942856

[30] Mautner L , Hoyos M , Dangel A , Berger C , Ehrhardt A , Baiker A . 2022. Replication kinetics and infectivity of SARS-CoV-2 variants of concern in common cell culture models. Virol J 19:76. doi:10.1186/s12985-022-01802-5 35473640 PMC9038516

[31] Zhao H , Lu L , Peng Z , Chen L-L , Meng X , Zhang C , Ip JD , Chan W-M , Chu AW-H , Chan K-H , Jin D-Y , Chen H , Yuen K-Y , To KK-W . 2022. SARS-CoV-2 Omicron variant shows less efficient replication and fusion activity when compared with Delta variant in TMPRSS2-expressed cells. Emerg Microbes Infect 11:277–283. doi:10.1080/22221751.2021.2023329 34951565 PMC8774049

